# *In vitro* evaluation of cancer-specific NF-*κ*B-CEA enhancer–promoter system for 5-fluorouracil prodrug gene therapy in colon cancer cell lines

**DOI:** 10.1038/sj.bjc.6603930

**Published:** 2007-08-07

**Authors:** X Guo, T R J Evans, S Somanath, A L Armesilla, J L Darling, A Schatzlein, J Cassidy, W Wang

**Affiliations:** 1 Oncology Group, Research Institute in Healthcare Science, School of Applied Sciences, University of Wolverhampton, Wolverhampton WV1 1SB, UK; 2 Cancer Research UK Beatson Laboratories, Centre for Oncology and Applied Pharmacology, University of Glasgow, Glasgow G61 1BD, UK; 3 Pharmacology Group, Research Institute in Healthcare Science, School of Applied Sciences, University of Wolverhampton, Wolverhampton WV1 1SB, UK; 4 The School of Pharmacy, University of London, London WC1N 1AX, UK

**Keywords:** NF-*κ*B, CEA, GDEPT, thymidine phosphorylase, colorectal cancer

## Abstract

Nuclear factor-kappa B (NF-*κ*B) is a transcription factor with high transcriptional activity in cancer cells. In this study, we developed a novel enhancer–promoter system, *κ*B4-CEA205, in which the basal carcinoembryonic antigen (CEA) promoter sequence (CEA205) was placed downstream of the four tandem-linked NF-*κ*B DNA-binding sites (*κ*B4). In combination with a *κ*B4 enhancer, the transcriptional activity of the CEA promoter was significantly enhanced (three- to eight-fold) in cancer cell lines but not in normal cells. In cancer cell lines, the transcriptional activity of *κ*B4-CEA205 was comparable with that of the SV40 promoter. We also constructed vectors in which the thymidine phosphorylase (TP) cDNA was under the control of CEA205, *κ*B4, *κ*B4-CEA205 and CMV promoters, respectively. TP protein and enzyme activity were detected at comparable levels in *κ*B4-CEA205- and CMV-driven TP cDNA-transfected cancer cell lines (H630 and RKO). The *κ*B4-TP and CEA205-TP-transfected cell lines, respectively, only demonstrated negligible and low levels of TP protein and enzyme activity. Both CMV- and *κ*B4-CEA205-driven TP cDNA transiently transfected cells were 8- to 10-fold sensitised to 5-fluorouracil (5-FU) prodrug, 5′-deoxy-5-fluorouradine (5′-DFUR), in contrast to only 1.5- to 2-fold sensitised by the *κ*B4- and CEA205-driven TP cDNA-transfected cells. The bystander killing effect of CMV- and *κ*B4-CEA205-driven TP cDNA-transfected cells was comparable. This is the first report that indicates that the NF-*κ*B DNA-binding site could be used as a novel cancer-specific enhancer to improve cancer-specific promoter activity in gene-directed enzyme prodrug therapy.

Lack of selectivity for malignant cells leading to dose-limiting toxicity is one of the major obstacles for the success of cancer chemotherapy. Gene-directed enzyme prodrug therapy (GDEPT) is potentially an elegant way to improve the therapeutic index of active anticancer drugs, but one of the major limitations for the clinical application of this approach is the lack of strong cancer-specific promoters (Aghi *et al*, 2000; Rooseboom *et al*, 2004).

Carcinoembryonic antigen (CEA) is not expressed in normal and overexpressed in epithelial cancer cells (Shively and Beatty, 1985). Four *cis*-acting DNA-binding elements mapped on the CEA promoter region within 300 bp upstream of CEA translation starting point, especially, the first three elements are essential for specific CEA transcription (Hauck and Stanners, 1995; Richards *et al*, 1995a). Utilisation of the CEA promoter in an adenovirus vector for cytosine deaminase (CD) gene expression improves the selectivity of 5′-deoxy-5-fluorocytidine (5-DFCR)/5-fluorouracil (5-FU) conversion in CEA-expressing tumours (Richards *et al*, 1995a; Lan *et al*, 1996a). However, the transcriptional activity of CEA in most cancer cell lines is 10- to 300-fold lower than that of CMV and RSV viral promoters (Lan *et al*, 1996b; Ueda *et al*, 2001). Even when using adenovirus to deliver CEA promoter-driven suicide gene *in vivo*, few reports have demonstrated sufficient antitumour effects. Although a 2.1-kb CEA enhancer can increase the CEA promoter activity, it is still highly dependent on the intrinsic level of CEA expression (Richards *et al*, 1995b; Nyati *et al*, 2002). The use of this CEA enhancer–promoter-based prodrug approach in cells with low levels of CEA expression cannot induce sufficient CD activity to convert 5-DFCR to 5-FU other than at very low levels (Richards *et al*, 1995b). As with other cancer-specific promoters, the low promoter activity is a major obstacle for using CEA promoter-based prodrug gene therapy *in vivo* and in clinical trials.

Nuclear factor-kappa B (NF-*κ*B) (Ephrussi *et al*, 1985) is a transcription factor, which is composed of five subunits (p50/p105, p52/p100, p65 (RelA), RelB and c-Rel) forming hetero- or homodimers. Nuclear factor-kappa B is normally retained in the cytoplasm as an inactive complex through the direct binding of the natural inhibitor of *κ*B (I*κ*B) (Karin, 1999). Nuclear factor-kappa B can be activated by cytokines, UV radiation, reactive oxygen species and anticancer drugs (Karin, 1999). All of these stimuli trigger phosphorylation and degradation of I*κ*B and release NF-*κ*B homo- or heterodimers, which are then translocated into nucleus and bound to DNA target site (*κ*B site) to influence downstream gene expression. *κ*B site is a strong *cis*-acting enhancer sequence possessing very weak transcriptional activity on its own. In cells with high NF-*κ*B activity, *κ*B enhancer specifically magnifies relevant promoter activity in a *cis*-acting manner (Ephrussi *et al*, 1985; Nelms *et al*, 1990). In a reporter vector, the enhancing activity of NF-*κ*B can be further improved by multitandem copies of *κ*B element located upstream of a weak promoter sequence. Compared with normal cells, most cancer cells possess high NF-*κ*B activity, which is produced by the cancer cells or the surrounding inflammatory cells. The NF-*κ*B activity in tumour tissues can be further induced by exposure to anticancer drugs, radiation or cytokines (Wang and Cassidy, 2003; Wang *et al*, 2003).

Capecitabine (CAP), a prodrug of 5-FU (Malet-Martino and Martino, 2002; Petty and Cassidy, 2004), has been approved as a first line therapeutic agent for colorectal cancer (CRC) chemotherapy. Capecitabine is converted into 5-FU *in vivo* by a three-enzyme-catalysed process. Capecitabine is converted to 5′-DFCR and then to 5′-deoxy-5-fluorouradine (5′-DFUR) by carboxylesterase and CD, respectively. Both conversions take place in liver and are not limiting steps for efficient conversion of CAP to 5-FU. The final and critical step is the conversion of 5′-DFUR to 5-FU by thymidine phosphorylase (TP). In comparison with normal cells, cancer cells express relatively higher levels of TP. The TP expression contrast between cancer and normal cells would potentially enable the enrichment of 5-FU in tumour cells, while reducing the exposure of normal tissues to 5-FU. However, TP is only detected in 24–66% (Evans *et al*, 2002) of CRC. Most tumours only express moderate levels of TP with 5-FU-resistant tumours expressing at even lower levels (Boyer *et al*, 2004). Consequently, insufficient conversion of 5′-DFUR into 5-FU in tumour tissues becomes the major limitation of CAP-based chemotherapy. Strong promoters such as CMV have been used to increase CD and TP expression in cancer cells and demonstrate enhanced cytotoxicity of 5′-DFCR and 5′-DFUR *in vitro* and *in vivo* (Huber *et al*, 1994; Patterson *et al*, 1995; Evrard *et al*, 1999; Ciccolini *et al*, 2001; Manome *et al*, 2001). Yet, the lack of specificity of these promoters limits the clinical development of GDEPT strategies.

Based on our understanding of the molecular biology of cancer, in this study, we used a non-virus expression system to develop a strong and highly cancer-specific NF-*κ*B-CEA enhancer–promoter system (*κ*B4-CEA205), which demonstrated high cancer-specific transcriptional activity. Transfection of TP cDNA driven by the NF-*κ*B-CEA enhancer–promoter cassette highly sensitised CRC cell lines to 5′-DFUR *in vitro*.

## MATERIALS AND METHODS

### Cell lines and chemicals

The human CRC cell lines HT29, HCT15, HCT116, LoVo, Caco2, DLD-1 and RKO were obtained from ATCC. H630 were generously provided by Professor PG Johnston (University of Belfast). Cancer cell lines were cultured in DMEM medium supplemented with 10% fetal calf serum (FCS), 50 U ml^−1^ penicillin and 50 *μ*g ml^−1^ streptomycin. Normal human mammary epithelial cells (HMEC, Clonetics, Rockland, ME, USA) were cultured in MEGM medium (Clonetics CC-3051) supplemented with BPE, hEGF, insulin and hydrocortisone. The human primary endothelial cells (HUVEC, Clonetics) were cultured in EBM-2 medium (Clonetics CC-3156) supplemented with hFGF-B, VEGF, hEGF and 2% FCS. The normal human nasal epithelial cells (HNEpC, PromoCell, Heidelberg, Germany) were cultured in the AECGM medium with SupplementMix (PromoCell C-39165). The primary cells were subcultured and maintained according to the instructed culture system from the suppliers. Human endothelial cell line EAhy926 was kindly provided by Dr Angel Armesilla (University of Wolverhampton, UK) and cultured in RPMI 1640 medium supplemented with 10% FCS, 50 U ml^−1^ penicillin and 50 *μ*g ml^−1^ streptomycin. Thymine, thymidine, 5′-DFUR and tetrazolium-based semiautomated colorimetric (MTT) were purchased from Sigma (Dorset, UK).

### Construction of luciferase and TP expression vectors

**Figure 1:**
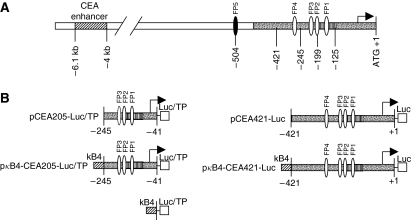
The schematic diagrams of the CEA promoter region and NF-*κ*B enhancer- and CEA promoter-related constructs used in this study. (**A**) The promoter region of CEA gene. FP: footprint site; basal CEA promoter region; essential CEA promoter region; arrow: transcriptional starting point; (**B**) NF-*κ*B enhancer- and CEA promoter-related constructs. Luc, luciferase cDNA; TP, thymidine phosphorylase cDNA.

The human CEA promoter cDNA (pDRIVE-hCEA) purchased from InvivoGen (San Diego, CA, USA) was subcloned by polymerase chain reaction (PCR). The CEA basal promoter sequences in this paper are numbered relative to the middle nucleotide of the first ATG (Schrewe *et al*, 1990). Two basal CEA promoter sequences (CEA205: −245 to −41 and CEA421: −421 to +1) were used to construct luciferase reporter vectors in combination with or without NF-*κ*B enhancer elements. As shown in Figure 1, both basal promoter sequences contain the essential CEA promoter region. CEA205 and CEA421 covered the first three and four *cis*-acting elements, respectively. The *Bgl*II and *Hin*dIII restriction sites were introduced into the forward and reverse primers, respectively. The primers used were as follows (the underlined: restriction sites). CEA205: forward 5′-GCGCAGATCTGAAAATAGAAGGGAAAAAAG-3′ and reverse 5′-GCGCAAGCTTGAGTTCCAGGAACGTTT-3′; CEA421: forward 5′-GCGCAGATCTAGAGCATGGGGAGACCCGGGA-3′ and reverse 5′-GCG CAAGCTTGGTCTCTGCTGTCTGCTCTGTC-3′. The PCR fragments were subcloned downstream of four tandem NF-*κ*B-binding sites (*κ*B4; 5′-GGGAATTTCC-3′ × 4) replacing the TATA-like promoter fragment into a commercially available pNF-*κ*B-Luc vector (BD Biosciences, San Jose, CA, USA). To produce the p*κ*B4-Luc vector without the essential promoter sequence, the TATA-like promoter sequence was removed by restriction enzyme cutting and the backbone plasmid DNA was blunt-ended using DNA polymerase Klenow fragment (Promega, Southampton, UK) and religated. To produce expression vectors without the NF-*κ*B enhancer, the *κ*B-binding sites in p*κ*B4-CEA205-Luc and p*κ*B4-CEA421-Luc were removed and the backbones religated as described above. Thymidine phosphorylase cDNA (pCMV6-XL5/TP) was purchased from Origene (Rockville, MD, USA). Thymidine phosphorylase cDNA was subcloned into pcDNA3 (Invitrogen, Paisley, UK) using *Eco*RI/*Xba*I to develop pcDNA3-TP in which TP cDNA is under the control of CMV promoter. To develop TP expression vectors for transient transfection, the luciferase cDNA in the above luciferase reporter gene expression vectors was replaced with TP cDNA derived from pcDNA3-TP by *Hin*dIII/*Xba*I cutting. For stable expression of TP in CRC cell lines, the CEA205-TP and *κ*B4-CEA205-TP fragments were subcloned into a pFastBacDual vector (Invitrogen, Paisley, UK) from which the p10 and polyhedrin promoters have been removed (pFastBacDualΔpromoter). The integrity of the sequences of the constructs was verified by DNA sequencing.

### TP-activity assay

The TP enzyme activity was assayed using the method published by Yoshimura *et al* (1990) with minor modification. Briefly, 10 *μ*l of total protein extracted in RIPA buffer containing 25 mM Tris-HCl, 0.1% SDS, 1% Triton X-100, 0.15 M NaCl, 1 mM EDTA and 1 × protease inhibitor mixture (Roche, East Sussex, UK) was mixed with 90 *μ*l of reaction buffer consisting of 10 mM thymidine, 10 mM KH_2_PO_4_ (pH 7.4). The reaction was incubated at 37°C for 2 h and terminated by addition of 400 *μ*l of 0.2 N NaOH. The concentration of thymine converted from thymidine was measured spectrophotometrically at 300 nm. The amount of thymine in the reaction was calculated from a standard curve plotted from known thymine concentrations. Thymidine phosphorylase activity was presented as nmol thymine/mg protein/hour.

### Western blot

Total protein was extracted in RIPA buffer. The lysate was centrifuged for 5 min in a microfuge and the supernatants retained. The protein (200 *μ*g/cell line) was electrophoresed through a 10% SDS-PAGE and transferred to a PVDF membrane (Millipore, Hertfordshire, UK) using a semi-dry electrophoretic transfer chamber (Millipore). Nonspecific binding was blocked by incubating the membranes for 1 h in TBS-T with 5% non-fat milk, which was also used to dilute primary (TP, Novocastra, Newcastle upon Tyne, UK, 1 : 250; CEA, Abcam, Cambridge, UK, 1 : 2000) and secondary (Amersham, Little Chalfont, UK; 1 : 5000) antibodies. The signal was detected using an ECL Western blotting detection kit (Amersham), and visualised by exposure to X-ray film.

### Total RNA isolation and RT–PCR

Cells (70% confluent) cultured in 25 cm flasks were harvested by trypsinisation. Total RNA was isolated using TRIzol reagent (Invitrogen, Paisley, UK) according to the manufacturer's protocols. The CEA mRNA expression levels in different cell lines were determined using the Access RT–PCR System (Promega, Southampton, UK) following the instruction of the supplier. The CEA-specific primers were designed from human CEA DNA sequence (GenBank ID: M17303). The forward (5′-CGC CAA AAT CAC GCC AAA TAA TAA-3′) and reverse (5′-ACC CCA ACC AGC ACT CCA ATC AT-3′) primers were used to amplify a 171 bp PCR product. The human housekeeping gene GAPDH (GenBank ID: XR018317) was used as the RNA loading control, which was amplified by the same RT–PCR system using the following primers, forward: 5′-CATGACAACTTTGGTATCGTG-3′; reverse: 5′-GTGTCGCTGTTGAAGTCAGA-3′. The RT–PCR amplification conditions were as follows. One cycle at 48°C for 45 min; 1 cycle at 94°C for 2 min; 35 cycles at 94°C for 30 s, 60°C for 30 s, 72°C for 1 min; 1 cycle at 72°C for 5 min. The RT–PCR products were separated on a 1% agarose gel and the bands visualised and photographed under UV light.

### MTT assay

For *in vitro* cytotoxicity analysis, the overnight-cultured cells (5000/well in 96-well flat-bottomed microtiter plates) were constantly exposed to 5′-DFUR for 72 h and then subjected to a standard MTT assay as previously described (Plumb *et al*, 1989). To test the effect of transient expression of TP on cytotoxicity of 5′-DFUR, cells were transiently transfected with TP expression vectors for 48 h and subjected to MTT assay. To determine the bystander effect, the pcDNA3-TP- or p*κ*B4-CEA205-TP-transfected cells were mixed with pcDNA3-transfected cells. The whole cell populations consisted of 0, 5, 10, 20, 40, 60 and 100% TP cDNA (pcDNA3-TP and p*κ*B4-CEA205-TP)-transfected cells, respectively, were exposed to 5′-DFUR and subjected to MTT analysis. The IC_50_s of 5′-DFUR were calculated.

### *In vitro* transfection

*Stable transfection* Colorectal cancer cell lines (1 × 10^6^/well) were cultured in six-well plates overnight and transfected with pFastBacDualΔpromoter/*κ*B4-CEA205-TP using Lipofectamine 2000 (Invitrogen, Pasley, UK) following the manufacturer's instructions. The pFastBacDual empty vector-transfected cells were used as a negative control. The successfully transfected clones were selected in G418 (1 mg ml^−1^). After TP enzyme activity assay and Western blotting analyses, the positive clones were selected and cultured in G418-containing medium to enlarge the cell population. Two positive clones were subjected to further analysis.

*Transient transfection* H630 and RKO cells (2 × 10^6^/flask) were cultured in 25 cm tissue culture flasks overnight and transfected with pcDNA3, pcDNA3-TP, p*κ*B4-TP, pCEA205-TP and p*κ*B4-CEA205-TP using Lipofectamine 2000 following the manufacturer's instruction. After 48 h transfection, the transfected cells were collected and split for MTT, Western blotting and TP activity assays, respectively.

### Luciferase reporter gene assay

All transfections were performed using Lipofectamine transfection reagent according to the instruction of the manufacturer (Invitrogen, Paisley, UK). Briefly, 5 × 10^4^/well cells were cultured in 24-well plates overnight. The luciferase reporter vectors (p*κ*B4-Luc, pCEA205-Luc, p*κ*B4-CEA205-Luc, pNF-*κ*B-Tal-Luc and pGL3-Basic; 0.8 *μ*g well^−1^) were co-transfected with 0.008 *μ*g well^−1^ pSV40-Renilla DNA, an internal control for normalisation of the transcriptional activity of the reporter vectors. Forty-eight hours after transfection, the cells were lysed and luciferase activities were determined using Dual Luciferase Assay reagents (Promega, Southampton, UK) according to the manufacturer's instructions. The luciferase activity in each well was normalised to pSV40-Renilla using the formula of Ln=*L*/*R* (Ln: normalised luciferase activity; L: luciferase activity reading and R: Renilla activity reading). The Ln was further standardised by the transcriptional activity of the pGL3-Basic using the formula of RLU=Ln/pGL3-basic (RLU: relative luciferase unit). All transfections were performed in triplicate and all experiments were repeated at least twice.

## RESULTS

### Background CEA and TP gene expression and the transcriptional activity of NF-*κ*B in CRC and normal cells

**Figure 2:**
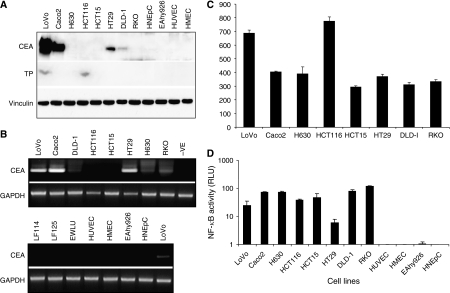
The expression of CEA and TP protein, CEA transcript and NF-*κ*B activity in CRC and normal cells. The CEA and TP protein expression levels were determined by Western blotting analysis (**A**). The transcription of CEA mRNA was detected by RT–PCR (**B**). Vinculin and GAPDH were used as loading control for Western blot and RT–PCR, respectively. The levels of TP enzyme activity in CRC cell lines were determined by TP enzyme assay (**C**). The transcriptional activity of NF-*κ*B was determined by luciferase reporter gene assay (**D**).

To evaluate the efficacy of NF-*κ*B-CEA enhancer–promoter system in 5′-DFUR prodrug based GDEPT, it is essential to determine the background status of TP (protein and enzyme activity), CEA (mRNA and protein) and NF-*κ*B (transcriptional activity) in cancer and normal cells. In a panel of eight CRC cell lines, only two (LoVo and HCT116) expressed the 55 kDa TP protein at moderate levels (25%). No TP protein expression was detected in the remaining six CRC and normal endothelial and epithelial cells (Figure 2A). The TP enzyme activity levels in the CRC cell lines were consistent with the TP protein expression levels (Figure 2C). The 180 kDa CEA protein was detected in four CRC cell lines (50%). Although the CEA mRNA was detected in most of the CRC cell lines (7/8, Figure 2B), it was only expressed at relatively high levels in three cell lines (LoVo, Caco2 and HT29) and at very low levels in the other four cell lines (DLD-1, HCT116, H630 and RKO). In contrast with cancer cell lines, no CEA protein and mRNA were identified in normal cells (Figure 2A and B). The transcriptional activity of NF-*κ*B in CRC and normal cells was also examined. High NF-*κ*B activity was detected in all eight CRC cell lines by luciferase assay. In contrast, the normal human cells only demonstrated negligible background levels of NF-*κ*B activity (Figure 2D).

### The transcriptional activity and specificity derived from different combinations of CEA basal promoters and NF-*κ*B enhancer

**Figure 3:**
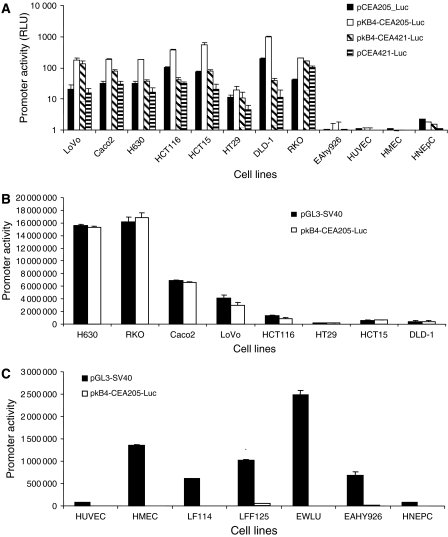
The transcriptional activity and specificity of CEA205 and CEA421 basal promoters in combination with or without *κ*B4 in CRC and normal human cell cultures. (**A**) The cells were co-transfected with luciferase expression vectors (pCEA205-Luc, p*κ*B4-CEA205-Luc, pCEA421-Luc, p*κ*B4-CEA421-Luc and pGL3-Basic) and Renilla expression vector (pRL3-SV40). The luciferase activity of each transfection was normalised by the Renilla reading. The luciferase activity is represented by the ratio of the specific promoter over the activity of pGL3-Basic. The column represents the mean of three measurements and the bar represents the s.d. (**B** and **C**) The cells were singly transfected with pGL3-SV40- or p*κ*B4-CEA205-Luc and the absolute luciferase activity in equal amount of protein from different transfections was compared.

To improve the transcriptional activity of CEA promoter and maintaining its cancer-targeting specificity, we took advantage of the high NF-*κ*B activity in CRC cell lines and designed a novel NF-*κ*B-CEA chimeric enhancer–promoter system (Figure 1). In this system, the basal CEA promoter sequences were placed downstream of the four directly linked NF-*κ*B DNA-binding sites (GGGAATTTCC) in tandem (*κ*B4). There are four *cis*-acting DNA-binding sites located within −303 to −143 base pairs of the CEA promoter region. The transcription factors Sp1, Sp1-like and USF bind to the first three DNA-binding sites and the transcription factors for the fourth element are still not clear (Hauck and Stanners, 1995). To develop an optimal enhancer–promoter system, we firstly compared the transcriptional activity and specificity of the luciferase reporter vectors driven by the basal CEA promoter regions covering the first three (CEA205) or four (CEA421) DNA-binding elements in combination with or without *κ*B4 enhancer (Figure 1). The transcriptional activities of the *κ*B4 enhancer, CEA basal promoters (CEA205 and CEA421) and chimeric enhancer–promoter elements (*κ*B4-CEA205 and *κ*B4-CEA421) were examined in CRC and normal human endothelial and epithelial cells. As showed in Figure 3A, both CEA205 and CEA421 basal promoters demonstrated selective transcriptional activity in all CRC cell lines but not in normal endothelial and epithelial cells. In combination with *κ*B4, the transcriptional activity of both CEA205 and CEA421 in cancer cell lines was enhanced. The *κ*B4 enhancer demonstrated a significantly greater enhancing effect on the CEA205 promoter than on the CEA421 promoter (Figure 3A). The *κ*B4 enhancer alone showed very low transcriptional activity in all tested cancer cell lines (Table 1). Only background transcriptional activity of CEA promoters, *κ*B4 enhancer and the chimeric *κ*B4-CEA enhancer–promoter cassette was detected in normal cells (Figure 3A and Table 1). Furthermore, we compared the transcriptional activity of *κ*B4-CEA205 with a strong SV40 virus promoter. To avoid the transcriptional interference between the SV40 promoter in pRL3-SV40 and pGL3-SV40, the CRC cell lines were singly transfected with p*κ*B4-CEA205 and pGL3-SV40. Equal amount of protein (10 *μ*g) from each transfection was subjected to luciferase assay. The transcriptional activity of *κ*B4-CEA205 enhancer–promoter system was highly comparable to that of the strong SV40 virus promoter in all CRC cell lines (Figure 3B). The SV40 promoter also demonstrated high transcriptional activity in a panel of seven normal human cell lines and primary cell cultures. In contrast, the transcriptional activity of p*κ*B4-CEA205 in normal cells was negligible (Figure 3C).

### 5′-DFUR cytotoxicity in CRC cell lines transiently transfected with a TP expression vector

**Figure 4:**
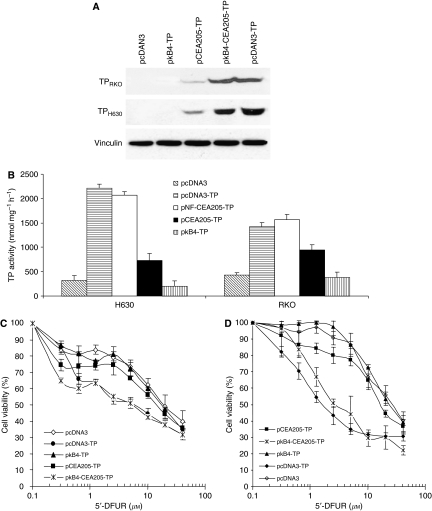
The TP enzyme status and cytotoxicity of 5′-DFUR in transiently transfected CRC cell lines. (**A**) Thymidine phosphorylase protein expression levels determined by Western blot. Vinculin: protein loading control. (**B**) Thymidine phosphorylase enzyme activity in different transfected cells. (**C** and **D**) The inhibitory effect of 5′-DFUR on the transiently transfected RKO (**C**) and H630 (**D**) cells. The curves were derived from three replicates and bars represent s.d.

Owing to the strong and cancer-specific transcriptional activity, the CEA205 promoter and *κ*B4-CEA205 enhancer–promoter cassette were subjected to further study. In the TP expression vectors, the TP cDNA was placed under the control of the CMV, CEA205, *κ*B4-CEA205 or *κ*B4 promoter. Two CRC cell lines (H630 and RKO) with low TP expression were transiently transfected with p*κ*B4-TP, pCEA205-TP, p*κ*B4-CEA205-TP, pcDNA3-TP or pcDNA3 empty vector. After 48 h transfection, the cells were harvested and subjected to different analysis. High levels of TP protein were detected in both pcDNA3-TP- and p*κ*B4-CEA205-TP-transfected cells. Only very low levels of TP protein were detected in the pCEA205-TP-transfected cells and no TP protein was detected by Western blotting analysis in the pcDNA3- and p*κ*B4-TP-transfected cell lines (Figure 4A). In line with protein expression levels, the pcDNA3-TP- and p*κ*B4-CEA205-TP-transfected cells also possessed high TP enzyme activity (Figure 4B). The TP activity and protein expression patterns were highly consistent one another in the transfected cells. The transiently transfected cells were then subjected to MTT analysis. In comparison with the control (pcDNA3 transfected), the p*κ*B4-CEA205-TP-transfected RKO and H630 cells were 7.6 and 10 times, respectively, more sensitive to the 5′-DFUR-induced cytotoxicity. The cytotoxicity of 5′-DFUR to the p*κ*B4-CEA205-TP- and pcDNA3-TP-transfected cell lines was comparable. In comparison with pcDNA3-TP- and p*κ*B4-CEA205-TP-transfected cells, the p*κ*B4-TP and pCEA205-TP transfection only slightly increased the cytotoxicity of 5′-DFUR to the CRC cell lines (Figure 4C and D and Table 2).

### The bystander effect of pFastBacDualΔpromoter/*κ*B4-CEA205-TP-transfected CRC cells on the neighbouring cells

**Figure 5:**
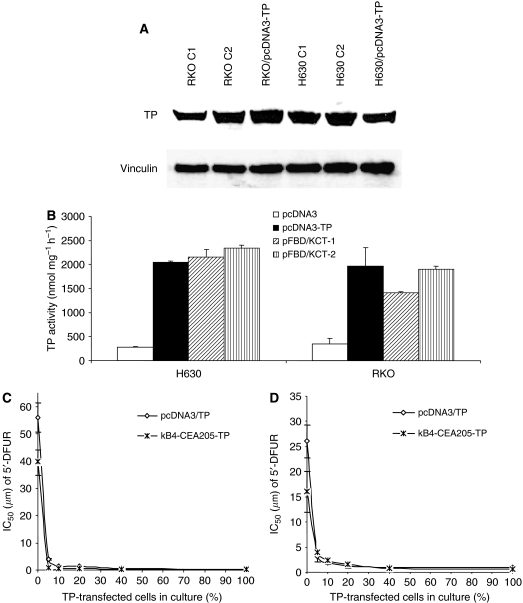
The TP protein (**A**) and enzyme activity (**B**) levels in the TP cDNA stably transfected cancer cell lines. pFBD/KCT-1 and pFBD/KCT-2: pFastBacDual/*κ*B4-CEA205-TP clone 1 and clone 2. Vinculin: protein loading control. The bystander killing effect of CMV- or *κ*B4-CEA205-driven TP cDNA transfection was compared in H630 (**C**) and RKO (**D**) cells. The curves were derived from three replicates and the bars represent s.d.

To produce cell lines for the bystander effect analysis, the H630 and RKO cell lines were stably transfected with pFastBacDualΔpromoter/*κ*B4-CEA205-TP (pFBD/KCT) or pcDNA3-TP. Two positively transfected clones were selected from each cell line for further study. The TP protein expression and TP enzyme activity in the pFBD/KCT-transfected cell lines were high and comparable with that in the pcDNA3-TP-transfected cells (Figure 5A and B). Furthermore, the cytotoxicity of 5′-DFUR to the pcDNA3-TP and pFBD/KCT stably transfected cell lines was determined by MTT analysis. The pFBD/KCT stable transfection significantly enhanced the cytotoxicity of 5′-DFUR to both RKO and H630 cell lines (over 28-fold). The IC_50_s of 5′-DFUR to the pFBD/KCT-transfected cells were similar to those of pcDNA3-TP-transfected cells (Table 3). Low transfection rate is still one of the major barriers for the success of cancer GDEPT in the clinic. It has been reported that the small portion of CMV promoter-driven TP-transfected cells can effectively convert 5′-DFUR into 5-FU, which can be released and performed cytotoxic effect onto the neighbouring cells (bystander effect) (Patterson *et al*, 1995). Figure 5C and D show that when pcDNA3-TP- or pFBD/KCT-transfected RKO or H630 cells composed only 5% of the whole-cell population, the sensitivity of H630 and RKO cell lines to 5′-DFUR was significantly enhanced. If the percentage of the transfected cells was further increased, the IC_50_s of 5′-DFUR in these cells were further reduced but the curves significantly levelled off. The bystander effect of pFBD/KCT- and pcDNA3-TP-transfected cells was comparable.

## DISCUSSION

As with other cancer-specific promoter systems, low transcriptional activity limits the clinical use of the CEA promoter in GDEPT. It has been reported that the transcriptional activity of CEA promoter is significantly improved by multimerisation of the essential CEA promoter region and/or fusing the CEA promoter with a CEA enhancer sequence. Although the transcriptional activity of CEA promoter is significantly improved by these modifications, all of these approaches are highly cell line and CEA activity dependent. The transcriptional activity of these modified promoter systems remains at very low levels in the low CEA-expressing cancer cells (Richards *et al*, 1995a; Nyati *et al*, 2002). High NF-*κ*B activity is a very common feature of cancers (Rayet and Gelinas, 1999). In this study, four linked NF-*κ*B-binding sites in tandem (*κ*B4) were exploited as a cancer-specific enhancer to improve the transcriptional activity of the CEA promoter. The enhancing effect of *κ*B4 on the transcriptional activity of two basal CEA promoters covering the first three (CEA205) and four (CEA421) DNA-binding sites on the CEA promoter region was examined. Both promoters demonstrated comparable transcriptional activity, which was further enhanced by *κ*B4 in CRC cell lines. In comparison with that on CEA421, the *κ*B4 enhancer demonstrated stronger enhancing effect on CEA205 promoter (Figure 3A). The luciferase assay showed that the transcriptional activity of *κ*B4-CEA205 was comparable with that of SV40 promoter (Figure 3B). Similar to other cancer-related genes, although CEA and NF-*κ*B are highly expressed in cancer cells, the low levels of both are still detectable in some normal tissues (Hammarstrom, 1999). Therefore, we also tested the potential transcriptional leakage of the NF-*κ*B-CEA enhancer–promoter system. In agreement with a previous report (Perkins *et al*, 1993), the NF-*κ*B-binding sites by itself, as an enhancer element, only possessed very low transcriptional activity. The transcriptional activity in cancer cell lines was highly increased by the combination of *κ*B4 and CEA promoter and meanwhile the cancer-targeting specificity was still remained (Figure 3A and C and Table 1). Our results demonstrated that *κ*B4 enhancer efficiently improved the transcriptional activity of CEA basal promoter without impairing its cancer specificity. The fidelity of the *κ*B4-CEA205 enhancer–promoter system was therefore improved by the increased transcriptional contrast between the cancer and normal cells.

In this study, TP/5′-DFUR was used as a model to test the efficacy of the *κ*B4-CEA205 enhancer–promoter system. Thymidine phosphorylase is a key enzyme for CAP, a 5-FU prodrug (Malet-Martino and Martino, 2002). As the TP activity in various cancers is highly heterogeneous, the clinical response of cancer patients to CAP remains relatively low (Malet-Martino and Martino, 2002). In this study, the TP protein expression was only detected in two CRC cell lines. Transient transfection of p*κ*B4-CEA205-TP and pcDNA3-TP resulted in comparable expression levels of TP protein in human CRC cell lines. In contrast, the pCEA205-TP-transfected cells only expressed TP protein at very low levels. The TP protein expression in these cell lines was reflected by the TP activity assay results. These results indicated that the transcriptional efficacy of *κ*B4-CEA205 was comparable with that of CMV strong virus promoter. In line with the TP protein and enzyme activity results, the MTT assay demonstrated that the cytotoxicity of 5′-DFUR to H630 and RKO cell lines was highly sensitised by the transfection of pcDNA3/TP or p*κ*B4-CEA205-TP (7.6 to 14.5-fold, Figure 4C and D and Table 2).

One of the major limitations for the clinical application of GDEPT is the low transfection rate *in vivo*, which can be overcome by bystander effect. Using strong virus promoters, it has been reported that the 5-FU derived from 5′-DFUR can diffuse from the transfected cancer cells to exert bystander killing effect on their neighbour cells in a gap junction-independent manner (Patterson *et al*, 1995; Kato *et al*, 1997). In this study, we also compared the bystander effect of pFBD/KCT- and pcDNA3/TP-transfected cancer cell lines. Figure 5C and D shows that the cytotoxicity of 5′-DFUR to RKO and H630 cell lines was significantly enhanced by the mixture of only 5% of pcDNA3/TP- or pFBD/KCT-transfected cells. The bystander effect was levelled off when further increasing the TP-transfected cells in the mixture. As well, the bystander effect of pcDNA3/TP and pFBD/KCT transfection was very similar in both cell lines.

The molecular mechanisms of enhancing effect of *κ*B4 on CEA promoter activity in this study were still not clear. Although by definition, enhancer is a DNA element, which efficiently regulates promoter activity distantly and orientation-independently (Banerji *et al*, 1983; Sassone-Corsi *et al*, 1983), the relationship between enhancer and promoter elements is far more complex. A cooperative interaction between NF-*κ*B and Sp1 has been demonstrated in human immunodeficiency virus long terminal repeat (HIV-1 LTR) promoter region (Nabel and Baltimore, 1987; Perkins *et al*, 1993). The cooperation between NF-*κ*B and Sp1 in the HIV-1 LTR is highly space and orientation-dependent. The promoter activity is significantly impaired by the insertion of DNA fragments between the *κ*B- and Sp1-binding sites. Mutation of the adjacent *κ*B and Sp1 DNA-binding sites completely abolishes the phorbol myristate acetate inducible transcriptional activity (Perkins *et al*, 1993). It was also reported that an Sp1-dependent promoter is distantly enhanced by an NF-*κ*B enhancer (Teferedegne *et al*, 2006). The cooperation between NF-*κ*B and Sp1 needs direct contact of both proteins and other transcription modulators (Perkins *et al*, 1993; Teferedegne *et al*, 2006). Chromatin modulation is also involved. Two Sp1 and Sp1-like DNA-binding sites are located on the CEA promoter region (FP2 and FP3) (Chen *et al*, 1995; Hauck and Stanners, 1995). Therefore, the interaction between NF-*κ*B and Sp1 may also play a role in our *κ*B4-CEA205 enhancer–promoter system. The *κ*B4 enhancer demonstrated higher enhancing effect on CEA205 than CEA421 in most CRC cell lines (Figure 3A). The distance between the *κ*B4 and the 5′ Sp1 in *κ*B4-CEA205 and *κ*B4-CEA421 fragments was 29 and 250 nt, respectively. The spatial rule may also partially apply to the enhancer–promoter systems in this study. Sp1 is a common element in cancer-specific promoters, for example CEA, survivin and hTERT (Chen *et al*, 1995; Hauck and Stanners, 1995; Li and Altieri, 1999; Takakura *et al*, 1999). Our unpublished data showed that *κ*B4 also improved the transcriptional activity of hTERT promoter. Therefore, investigation of the relationship between *κ*B4 and Sp1 might provide evidence for using *κ*B4 to improve the transcriptional activity of other cancer-specific promoters.

The lack of strong cancer-specific promoter system remains one of the major obstacles for the clinical success of GDEPT. This is the first *in vitro* study using NF-*κ*B DNA-binding site as a cancer-specific enhancer element to improve the cancer-specific transcriptional activity of CEA promoter. The *in vivo* investigation needs to be carried out to use this enhancer system for GDEPT approach.

## Figures and Tables

**Table 1:** Transcriptional activities of *κ*B4, CEA205 and *κ*B4-CEA205 in CRC and normal cells

	**LoVo**	**Caco2**	**H630**	**HCT116**	**HCT15**	**HT29**	**DLD-1**	**RKO**	**EAhy926**	**HUVEC**	**HMEC**	**HNEpC**
p*κ*B4-Luc	5.6±0.7	13.0±4.5	13.0±2.7	14.0±2.1	13.8±4.0	2.3±0.7	34.4±3.4	12.2±0.3	1.0±0.5	1.0±0.08	1.0±0.08	0.7±0.003
pCEA205-Luc	20.4±8.3	31.3±5.1	31.3±4.7	101.1±5.2	72.8±5.3	11.3±2.0	191.3±18.9	42.0±2.3	1.1±0.6	1.1±0.02	1.1±0.02	2.3±0.01
p*κ*B4-CEA205-Luc	180.6±23.9	183.9±16.9	183.9±2.6	379.3±21.4	558.3±83.8	19.6±6.4	993.6±68.1	207.4±3.8	1.0±0.003	0.95±0.003	0.95±0.003	1.8±0.02

The data represent mean relative luciferase units from three replicates±s.d.

**Table 2:** The cytotoxicity of 5′-DFUR on the transiently transfected CRC cell lines

	**pcDNA3**	**pcDNA3/TP**	**p*κ*B4-TP**	**pCEA205-TP**	**p*κ*B4-CEA205-TP**
H630	33.4±3.2	2.3±1.0	28.3±1.9	25.1±0.8	3.3±1.2
RKO	33.6±9.1	3.8±2.5	20.7±2.5	15.6±1.1	4.4±0.2

The data represent the mean IC_50_ (*μ*m) of 5′-DFUR from three replicates±s.d.

**Table 3:** The cytotoxicity of 5′-DFUR on the stably transfected CRC cell lines

			**pFastBacDualΔpromoter/*κ*B4-CEA205-TP**	
	**pcDNA3**	**pcDNA3/TP**	**C1**	**C2**
H630	27.7±12.9	0.94±0.54	0.96±0.02	0.94±0.03
RKO	28.3±13.2	1.16±0.71	0.90±0.07	1.00±0.06

The data represent the mean IC_50_ (*μ*m) of 5′-DFUR from three replicates±s.d. C1 and C2: clones 1 and 2.
